# One health approach to *Balantioides coli*: Molecular and metabarcoding evidence of zoonotic transmission

**DOI:** 10.1371/journal.pone.0338487

**Published:** 2026-01-05

**Authors:** Camila Souza Carvalho Class, Pedro Mendes de Souza, Renan de Souza Ferreira, Ingrid da Silva Reis, Laís Lisboa Corrêa, Breno Torres da Silva, Gabriella Ribeiro Vaz da Costa, Fabiana Batalha Knackfuss, Roberto Júnio Pedroso Dias, Alynne da Silva Barbosa

**Affiliations:** 1 Department of Microbiology and Parasitology, Biomedical Institute, Fluminense Federal University, Niterói, Rio de Janeiro, Brazil; 2 Laboratory of Protozoology, Institute of Biological Sciences, Federal University of Juiz de Fora, Juiz de Fora, Minas Gerais, Brazil; 3 Faculty of Veterinary Medicine, Centro Universitário Serra dos Órgãos (UNIFESO), Teresópolis, Rio de Janeiro, Brazil; 4 Laboratory of Statistics, UNIGRANRIO (AFYA University), Duque de Caxias, Rio de Janeiro, Brazil; University of Ghana, GHANA

## Abstract

*Balantioides coli* is a neglected zoonotic protozoan, with pigs recognized as its main reservoirs. In tropical countries such as Brazil, information on balantidiasis in occupationally exposed human populations remains limited. In this context, this study investigated the occurrence of *B. coli* and other intestinal parasites in pig farmers, their families, and pigs from the same farms, while also exploring associations with sanitary conditions and the gut microbiota. Fecal samples from 47 humans and 15 pigs from family-owned and industrial farms in Rio de Janeiro and Minas Gerais, Brazil, were analyzed. General and sanitary information was obtained through structured questionnaires. Samples were examined by microscopy, and *B. coli* was identified by PCR targeting ITS1–5.8S–ITS2, followed by Sanger sequencing. The gut microbiota was characterized using 18S and 16S rRNA (V3/V4) molecular markers for metabarcoding. Among the 47 participants, seven (14.9%) tested positive for *B. coli* nucleotide sequences in their fecal material, with most of them (71.4%) being adult males directly involved in pig handling. Phylogenetic analysis revealed genetic variants A0, B0, and atypical strains shared between humans and pigs from the same farm. Eukaryotic parasites (*B. coli*, *Blastocystis* spp., *Entamoeba* spp.) and prokaryotic groups (Firmicutes, Bacteroidota, and potentially pathogenic bacteria) were also shared across hosts, and two human–pig pairs (H31–129F and H70–565F) exhibited highly similar microbial profiles. This study provides the first molecular evidence of *B. coli* in pig farmers in Brazil, reinforcing the epidemiological role of pigs in zoonotic transmission. These findings highlight the need for improved sanitation, consistent use of personal protective equipment, and continuous molecular surveillance under a One Health perspective to minimize transmission of this parasite.

## Introduction

*Balantioides coli* (Malmsten, 1857), formerly known as *Balantidium coli*, is a unicellular eukaryote belonging to the phylum Ciliophora [1]. To date, *B. coli* is the only ciliated protozoan known to infect humans and preferentially colonizes the cecum and colon. Pigs and non-human primates are considered the main natural reservoirs of this parasite, playing a key role in its zoonotic transmission [1]. However, *B. coli* has also been identified in a wide range of hosts, including wild boars, cattle, buffaloes, sheep, goats, camels, equids, rodents, rheas, and ostriches [2,3].

The parasitic infection caused by *B. coli* is referred to as balantidiasis, also known as balantidian dysentery, ciliate dysentery, or balantidiosis. Transmission occurs predominantly via the fecal–oral route, usually through ingestion of water or food, such as fruits and vegetables, contaminated with cysts. Infection can also result from direct contact with the feces of infected hosts, and is more frequent where close contact occurs between humans and animals, particularly pigs [1,4].

In humans, *B. coli* infections may be asymptomatic; acute, presenting dysentery of varying severity that can progress to fulminant disease; or chronic, ranging from diarrhea with abdominal discomfort to more severe invasive forms with dysentery and mucosal ulceration [5,6]. Although the cecum and colon are the primary sites of *B. coli* colonization, extraintestinal infections have been reported, affecting the peritoneal cavity, liver, gallbladder, genitourinary tract, lungs, eyeball, and even vertebrae [7–9]. Several factors may influence host susceptibility to infection, including host nutritional status, *B. coli* strain virulence, gut microbiota composition, concurrent enteric infections, and parasite load [4,10].

The pathogenicity of *B. coli* in pigs remains a matter of debate, with no clear consensus on whether the organism behaves primarily as a commensal or a parasite, nor on its true pathogenic potential [11–13]. Even so, clinical outcomes in pigs can mirror those described in humans, ranging from silent infections to acute or chronic balantidiasis [12,13].

*Balantioides coli* is a neglected parasite, and its epidemiology in humans remains poorly understood, with most data limited to isolated reports of symptomatic cases [4]. The majority of human balantidiasis records are concentrated in Asia, South America, and Africa, notably India, Brazil, Venezuela, and Ethiopia [4]. Balantidiasis is more prevalent in tropical and humid regions, where environmental conditions favor cyst survival and dissemination [6].

In Brazil, data on the prevalence of *B. coli* in occupationally exposed populations are scarce, particularly among rural workers who handle pigs. Close and prolonged contact with these natural reservoirs, combined with often inadequate farm sanitation, facilitates zoonotic transmission [6]. Nevertheless, epidemiological studies to date have not detected *B. coli* in this high-risk group [14–16].

Recent studies have described characteristic gut microbiota patterns associated with colonization by gastrointestinal parasites, including *Giardia duodenalis*, *Blastocystis* spp., *Entamoeba* spp., and *Ascaris* spp. [17]. These findings suggest that interactions between intestinal parasites and the bacterial microbiota may influence both pathogenesis and host immune responses, highlighting the importance of integrated approaches to understand these complex processes. In this context, microbiome analyses have benefited from metabarcoding techniques that enable simultaneous characterization of multiple taxonomic groups in biological samples. Among available platforms, Illumina is widely used owing to its high accuracy, low error rates, and deep sequencing coverage, enabling the detection of low-abundance organisms in complex matrices such as feces [18].

Moreover, this technology allows for the parallel generation of large volumes of data, supporting both microbial diversity analyses and the simultaneous detection of eukaryotic and prokaryotic organisms. These features make Illumina particularly effective in parasitological studies, contributing to the detection of genetic variants, rRNA region mapping, intragenomic variability investigations, and the exploration of relationships among different taxa [18]. However, investigations on *B. coli* remain limited. Observations in weaned piglets with diarrhea have revealed high numbers of *B. coli* trophozoites associated with abundant, potentially pathogenic bacteria, including *Escherichia coli*, *Shigella*, and *Campylobacter* [19,20].

Although human contact with pigs poses a risk for zoonotic transmission of *B. coli*, no study has yet identified this parasite in fecal samples from rural pig farmers in Brazil using molecular methods, underscoring a critical knowledge gap. This study is based on the hypothesis that close contact between farmers and pigs facilitates direct zoonotic transmission of *B. coli* and may be associated with similarities in the gut microbiota between hosts. Accordingly, the objectives were to (i) determine the occurrence of *B. coli* and other intestinal parasites in fecal samples from pig farmers and their family members using microscopic and molecular tools; (ii) correlate parasite detection with general and sanitary information; (iii) genetically characterize the parasite; and (iv) explore the taxonomic composition of the eukaryotic and prokaryotic microbiota in feces from both farmers and their pigs.

## Materials and methods

### Study site, population, questionnaire, and fecal sample collection

The study was conducted on family and industrial pig farms located in multiple municipalities across the states of Rio de Janeiro and Minas Gerais, Brazil. A total of 15 farms were included, of which 11 were family-ownedand 4 were industrial.

In the metropolitan region of Rio de Janeiro, two farms were included in each of the municipalities of Rio Bonito and Maricá, and one farm each in Itaboraí, Tanguá, and Silva Jardim. Three farms were included in the mountain region (two in Cachoeiras de Macacu and one in Nova Friburgo). Two farms were included in the coastal lowlands (Casimiro de Abreu and Saquarema), and one in the Médio Paraíba region (Pinheiral). Due to the limited number of industrial farms in Rio de Janeiro, two additional farms from neighboring municipalities in Minas Gerais (Rio Pomba and Barbacena) were included.

All participants either resided on or worked at pig farms. The study included pig handlers (n = 36) and their family members (n = 11), recruited through convenience sampling between July 3, 2023, and July 30, 2024. Written informed consent was obtained from all adult participants. For minors, assent forms were completed with authorization from their parents or legal guardians. Individuals who did not provide formal consent or who did not submit fecal samples were excluded from the study.

Participants who signed the informed consent completed a semi-structured questionnaire designed to collect general and sanitary -related information. The questionnaire comprised 23 open- and closed-ended questions, which were answered verbally and individually by each participant. For minors (children and adolescents), assent was provided by the participants, while parents and/or legal guardians signed the informed consent form and were responsible for completing the questionnaire. Responses were recorded in an online form (Jotform®) by the research team to minimize misinterpretation and issues related to visual impairment or illiteracy. Each participant was assigned a unique identification number to preserve anonymity.

After completing the questionnaire, study participants received kits for fecal sample collection. Each kit contained two collection containers: one without chemical preservative, which was stored under refrigeration (4°C), and another containing 70% ethanol, which could be kept at room temperature. Additionally, the kit included two wooden spatulas and a technical guide with detailed instructions on sample collection and storage. Verbal instructions on collection procedures were also provided at the time of kit delivery. Collections were scheduled in advance to ensure samples were refrigerated for no longer than two days.

Pig fecal samples were collected either directly from the rectal ampulla (using a glycerin-lubricated palpation glove) or immediately after spontaneous defecation. Following collection, all samples were individually labeled with identification numbers.

All human and pig fecal samples were placed in insulated containers and transported to the Parasitology Laboratories, Biomedical Institute, Federal Fluminense University, Niterói, Rio de Janeiro, Brazil.

### Laboratory and microscopic parasitological analyses

Human samples were processed separately from pig samples to avoid cross-contamination. Accordingly, human samples were processed in dedicated rooms, using commercial kits and other reagents used exclusively for these samples. Details on the collection and processing of pig fecal samples have been published previously [21].

In the laboratory, human fecal samples were subjected to on the same day to direct examination, followed by qualitative coproparasitological methods, including Lutz’s sedimentation [22] and Sheather’s centrifugal flotation [23] modified by Huber et al. [24], and the quantitative FLOTAC technique [25]. Slides from each method and the FLOTAC chambers were examined under an Olympus® BX41 light microscope at 100 × magnification, with confirmation at 400× when necessary.

### DNA extraction, polymerase chain reaction and phylogeny analyses

Fecal material from one pig per farm showing ciliated eukaryotic stages (cysts and trophozoites), along with all fecal samples from pig farmers and their family members, were subjected to DNA extraction. For DNA extraction from fecal samples, 200 mg of each sample were processed using the QIAamp Fast DNA Stool Mini Kit (Qiagen®). All extraction steps followed the manufacturer’s instructions, with two modifications suggested by a previously published protocol [26].

Extracted DNA was amplified by polymerase chain reaction (PCR) using the Hot Start Platinum Master Mix (Invitrogen®) and primers B58D (5’ GCTCCTACCGATACCGGGT 3’) and B58RC (5’ GCGGGTCATCTTACTTGATTTC 3’), which amplify the ITS1–5.8S–ITS2 region of ciliates, generating fragments of approximately 500 bp. Amplification followed the protocol described by Ponce-Gordo et al. [27]. A total of 5 µL of extracted DNA from each sample were used in the reaction. PCR products were visualized on a 1.5% agarose gel stained with GelRed® (Biotium®) and bromophenol blue (LGC®). PCR products were purified using ExoSAP-IT (Invitrogen®) and sequenced on an ABI 3730xL automated sequencer at the IOC/Fiocruz platform. Negative controls (ultrapure water) were included in all DNA extraction and PCR steps.

The ITS1–5.8S–ITS2 sequences obtained by Sanger sequencing were edited using ChromasPro® v.1.7.5 and incorporated into a sequence database retrieved from GenBank, comprising 255 sequences, including 243 additional *Balantioides coli* sequences and a set of 12 sequences from *Balantidium entozoon* (JQ408693.1, JQ408694.1)**,** *Buxtonella sulcata* (KP016715.1, KP016716.1, JQ073387.1, JQ073386.1)**,** *Buxtonella*-like (JQ073341.1, JQ073369.1, JQ073381.1, JQ073384.1, JQ073385.1), and *Troglodytella abrassarti* (EU680314.1), which were included as outgroups.

For phylogenetic analyses, we selected sequences identified as *B. coli* corresponding to the ITS1–5.8S–ITS2 region, with a length greater than 500 bp and containing variant descriptions. Sequences with ambiguous variant annotations were excluded from the dataset.

The resulting dataset was aligned using MAFFT [28] and subsequently processed with Gblocks [29] to remove poorly aligned regions, yielding a final alignment of 401 bp. The final dataset was visually inspected in SeaView (v.5.0.5) to ensure quality. Maximum likelihood phylogenetic reconstruction was performed using the IQ-TREE Web Server [30] with 1,000 bootstrap pseudoreplicates, employing the TN + F + G4 substitution model, which was automatically selected by the software.

### Library preparation and metabarcoding analyses

DNA extracted from pigs (n = 15) and humans (n = 13) that showed molecular evidence of Ciliophora by PCR was sent to Genone® for sequencing on the Illumina HiSeq 2500 platform. Amplicon libraries were prepared by PCR amplification of the V3–V4 region of the bacterial and archaeal 16S rRNA gene using primers 341F (CCTAYGGGRBGCASCAG) and 806Rb (GGACTACNNGGGTATCTAAT) [31], and by amplification of the V4 region of the eukaryotic 18S rRNA gene using primers TAReuk454FWD1 (CCAGCASCYGCGGTAATTCC) and TAReukREV3 (ACTTTCGTTCTTGATYRA) [32].

Initial quality control of Illumina sequencing results was performed by the sequencing provider. Subsequent analyses were then performed in QIIME 2 software [33]. Raw paired-end sequences had adapters removed and were demultiplexed using the q2-cutadapt [34] and q2-demux plugins, respectively. Reads were merged, denoised, and dereplicated using the q2-dada2 plugin [35]. Reads with a forward–reverse overlap of <20 bp and reads shorter than 225 bp were discarded. Chimeric sequences were also identified and removed using the “consensus” method implemented in q2-dada2. Amplicon sequence variants (ASVs) recovered and separated by q2-dada2 were taxonomically assigned using the QIIME2 feature-classifier (Sklearn) trained on the PR2 database [36]. ASVs identified as potentially pathogenic were retained for downstream diversity analyses*.*

### Data analyses

Results for *Balantioides coli* were interpreted through the integration of microscopic and molecular techniques. Molecular detection was performed by PCR targeting the ITS1–5.8S–ITS2 fragment, followed by Sanger sequencing, to confirm the parasite’s presence and to characterize its genetic variants.

The frequency of the parasite was calculated by dividing the number of positive samples by the total number of samples collected, and the results were expressed as percentages. All data, including questionnaire results, were stored in Microsoft Excel. Qualitative information collected from the questionnaire was tabulated and presented descriptively as percentages, with the most frequently reported responses highlighted in tables.

The nucleotide sequences of *B. coli* obtained by Sanger sequencing were compared phylogenetically with other sequences deposited in GenBank, considering country of origin, host species, and reported genetic variant.

Relative abundance analyses and Venn diagrams were used to explore the taxonomic composition of the overall eukaryotic and prokaryotic microbiota, as well as potentially pathogenic parasites present in the fecal samples of rural pig farmers and their family members raising pigs infected with *B. coli*. These plots were generated using the Python libraries matplotlib [37], plotnine [38], and pandas [39].

Microbiota dissimilarity and diversity were assessed using principal coordinates analysis (PCoA) based on Bray–Curtis distances and Weighted UniFrac metrics. Bray–Curtis considers taxon richness and abundance, whereas Weighted UniFrac also incorporates phylogenetic relatedness. PERMANOVA (999 permutations) was performed, considering p ≤ 0.05 as statistically significant. All analyses were performed using the q2-diversity plugin in QIIME 2 [33].

### Ethical considerations

This study was approved by the Human Research Ethics Committees of the participating institutions: Federal Fluminense University (UFF) under CAAE number 63709022.0.0000.5243, protocol no. 5.966.227; Federal Institute of Education, Science and Technology of Rio de Janeiro (IFRJ) under CAAE number 63709022.0.3003.5268, protocol no. 6.045.672; and Federal Institute of Education, Science and Technology of Minas Gerais (IFMG) under CAAE number 63709022.0.3002.5588, protocol no. 6,072,109.

In addition, the study was approved by the Animal Use Ethics Committee of Fluminense Federal University (protocol no. 8394191222), as well as by the Animal Use Ethics Committees of the participating Federal Institute of Education, Science, and Technology Rio de Janeiro (protocol no. 03/2022) and Minas Gerais (protocol no. 02/2023).

## Results

### Participant profile and microscopic parasitological results

A total of 47 individuals participated in this study, including farm owners, employees, and their family members. Of these, 36 were pig handlers and 11 were family members residing on the farm premises. Twenty participants worked on industrial farms, and 27 on family farms. Most participants (82.9%) were adult males who directly handled pigs as primary caretakers. In addition, 61.7% reported living on the farm premises and, 80.8% had previously undergone the stool examinations, with 29.7% reporting annual testing. Self-reported symptoms compatible with parasitic infections—such as abdominal pain, diarrhea, visible worms, and blood in stool—ranged from 4.2% to 23.4%. More than 80% reported previous use of antiparasitic drugs; however, over half could not recall the frequency of use (Table 1).

**Table 1:** Positivity for gastrointestinal parasites, with emphasis on *Balantioides coli*, based on molecular and microscopic parasitological techniques, along with general sanitary information from fecal samples of pig farmers on family and industrial farms in the states of Rio de Janeiro and Minas Gerais, Brazil.

Information	Number of participants	*Balantioides coli*	Other parasites*
**Age**			
< 18 years	8 (17.02%)	1 (14.29%)	1 (33.33%)
≥ 18 years	39 (82.98%)	6 (85.71%)	2 (66.67%)
**Sex**			
Male	35 (74.47%)	5 (71.43%)	2 (66.67%)
Female	12 (25.53%)	2 (28.57%)	1 (33.33%)
**Residence in the peridomicile of the pig farm**			
Yes	29 (61.70%)	3 (42.86%)	3 (100%)
No	18 (38.30%)	4 (57.14%)	0
**Fecal examination**			
Yes	38 (80.85%)	4 (57.14%)	2 (66.67%)
No	7 (14.89%)	2 (28.57%)	0
Unknown	2 (4.26%)	1 (14.29%)	1 (33.33%)
**Frequency of fecal examination**			
Every 6 months	7 (14.89%)	0	0
Once a year	14 (29.79%)	2 (28.57%)	1 (33.33%)
Not applicable	26 (55.32%)	5 (71.43%)	2 (66.67%)
**Antiparasitic drugs**			
Yes	40 (85.11%)	4 (57.14%)	3 (100%)
No	7 (14.89%)	3 (42.86%)	0
**Frequency of antiparasitic drug use**			
Every 6 months	8 (17.02%)	0	1 (33.33%)
Once a year	13 (27.66%)	3 (42.86%)	1 (33.33%)
Not applicable	26 (55.32%)	4 (66.67%)	1 (33.33%)
**Abdominal pain**			
Yes	11 (23.40%)	1 (14.29%)	0
No	36 (76.60%)	6 (85.71%)	3 (100%)
**Diarrhea**			
Yes	8 (17.02%)	2 (28.57%)	0
No	39 (82.98%)	5 (71.43%)	3 (100%)
**Detection of intestinal worms in stool**			
Yes	2 (4.26%)	0	1 (33.33%)
No	45 (95.74%)	7 (100%)	2 (66.67%)
**Detection of blood in stool**			
Yes	3 (6.4%)	1 (14.29%)	0
No	44 (93.61%)	6 (85.71%)	3 (100%)
**Type of drinking water at home**			
Treated municipal water	18 (38.30%)	2 (28.57%)	0
Untreated or non-potable water	29 (61.70%)	5 (71.43%)	3 (100%)
**Vegetable consumption**			
Yes	42 (89.36%)	7 (100%)	3 (100%)
No	5 (10.64%)	0	0
**Vegetable hygiene**			
Washed mechanically with water and/or other methods	34 (72.34%)	7 (100%)	3 (100%)
Use of chlorine, vinegar, or lemon	5 (10.64%)	0	0
Unknown	3 (6.38%)	0	0
Not applicable	5 (10.64%)	0	0
**Home gardens**			
Yes	27 (57.45%)	4 (57.14%)	2 (66.67%)
No	20 (42.55%)	3 (42.86%)	1 (33.33%)
**Type of fertilizer used in home gardens**			
Pig feces/manure	5 (10.64%)	0	2 (66.67%)
Manure from other animal species	7 (14.89%)	1 (14.29%)	0
No fertilizer used	15 (31.91%)	1 (14.29%)	0
Not applicable	20 (42.55%)	5 (71.42%)	1 (33.33%)
**Direct contact with pigs**			
Yes	42 (89.36%)	6 (85.71%)	2 (66.67%)
No	5 (10.64%)	1 (14.29%)	1 (33.33%)
**Uses personal protective equipment when handling pigs**			
Yes	35 (74.47%)	5 (71.43%)	1 (33.33%)
No	7 (14.89%)	1 (14.29%)	1 (33.33%)
Not applicable	5 (10.64%)	1 (14.29%)	1 (33.33%)
**Change of clothing after handling pigs**			
Yes	31 (65.96%)	5 (71.43%)	1 (33.33%)
No	11 (23.40%)	1 (14.29%)	1 (33.33%)
Not applicable	5 (10.64%)	1 (14.29%)	1 (33.33%)

*Hookworm eggs: *Necator americanus* and/or *Ancylostoma duodenale*; cysts and/or vegetative forms of protozoa: *Blastocystis* spp.

Untreated water sources, including wells and springs, were the most frequently reported (61.7%). Regarding vegetables, 72.3% of participants consumed them after washing with water or with solutions containing chlorine, vinegar, or lemon, and 57.4% maintained vegetable gardens on their properties. Some individuals reported using animal manure, including pig manure, to fertilize crops. More than 65% of participants reported using personal protective equipment when handling animals, and changing clothes after farm work (Table 1).

Three individuals tested positive by microscopic coproparasitological techniques: two harbored hookworm eggs (8–16 eggs/g feces), and one showed vacuolar forms of *Blastocystis* spp. All positive individuals were family members residing on a farm in Cachoeiras de Macacu, Rio de Janeiro. No forms compatible with the phylum Ciliophora were observed in participant samples.

### Nucleotide sequences and phylogenetic analysis of *B. coli* in rural workers in association with general and sanitary data

PCR amplification targeting the ITS1–5.8S–ITS2 region detected DNA of the phylum Ciliophora in fecal samples from 13 pig farmers and their family members. Of these, seven individuals yielded nucleotide sequences suitable for interpretation, all confirmed as *B. coli*, indicating that these individuals (7/47; 14.9%) carried parasite sequences in their feces. Four of these originated from caretakers on industrial farms and three from family-owned farms, all located in Rio de Janeiro.

**Fig 1:**
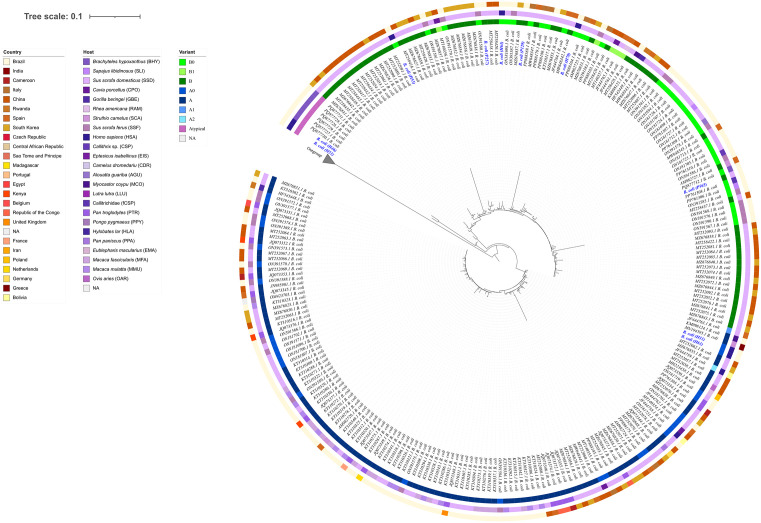
Maximum likelihood phylogeny of ciliates based on ITS sequences. Sequences of ciliates obtained in this study (highlighted in blue) and reference sequences from different countries, hosts, and genetic variants. The concentric rings indicate: (i) country of origin (outermost ring), (ii) host species (middle ring), and (iii) genetic variant (innermost ring). Scale bar: 0.1 substitutions per site.

Phylogenetic analysis of the sequences obtained in this study (Fig 1) revealed a distribution across distinct clades, indicating high genetic diversity. Three sequences from human samples (H53, H63, and H70) from an industrial farm in Nova Friburgo belonged to the B0 genetic variant, with identities of 97.2%–100% compared with GenBank references. Despite sharing the same variant, these sequences did not cluster within the same clade (Fig 1). In contrast, the A0 variant was detected in one human sample (H61) from the same farm and in a caretaker from a family-owned farm (H31), with identities of 98.6%–99.1%. Two additional sequences from pig handlers from family-owned farms (H46 and H51) were classified as atypical and clustered within a clade closely related to sequences from a neotropical primate (Fig 1).

All pig samples analyzed—one per farm, totaling 15 animals—yielded PCR amplification products for the ITS1–5.8S–ITS2 region. The 15 sequences generated were identified as *B. coli*, with identities of 97.8%–100% compared with GenBank references. Among these, three were classified as the A0 variant, while the remaining 12 belonged to the B0 variant. Notably, this variant was the only one identified in pigs from farms where *B. coli* sequences were also detected in humans (Fig 1). All sequences obtained in this study were deposited in GenBank with accession numbers as described in S1 Table.

Among the seven individuals positive for *B. coli* by PCR and confirmed by Sanger sequencing, most were male (71.4%), adults over 18 years (85.7%), and directly involved in pig husbandry (85.7%). More than half (=40%) reported living on the farm premises, had undergone previous testing for intestinal parasites, and had prior use of antiparasitic drugs. However, only two participants reported undergoing annual stool examinations, and most of them could not recall the frequency of antiparasitic drug use (Table 1).

Clinical symptoms compatible with parasitic infections were reported by some participants: 28% experienced diarrhea, and one reported abdominal pain with blood in the stool. None observed worms in their feces. More than 70% of positive participants reported consuming untreated water obtained from springs or artesian wells. All reported consuming vegetables after washing with water and additives such as chlorine, vinegar, or lemon, although only 57% maintained home gardens. Regarding fertilizer use, five participants (70%) could not provide this information (Table 1).

More than 70% of positive participants reported using personal protective equipment during pig handling, including boots, closed shoes, and long pants. One participant, however, reported handling animals without any protective measures. Similarly, 70% reported changing work clothing after animal contact (Table 1).

### Fecal samples from rural pig producers and pigs positive for *B. coli*: Comparison of unicellular eukaryote microbiota and other co-occurring taxa

Overall, the 13 human fecal samples that showed bands compatible with the phylum Ciliophora by polymerase chain reaction—originating from family farms in Saquarema and Silva Jardim, and from an industrial farm in Nova Friburgo (RJ)—as well as one positive pig sample from the same farms, were subjected to metabarcoding analysis of the V4 region of the 18S rRNA gene.

The initial processing generated a dataset comprising 1,170,330 reads. After primer removal and processing in QIIME2 software, the number of reads was reduced to 985,842. These reads were clustered into 628 amplicon sequence variants (ASVs). They were classified into 15 phyla, 62 classes, 139 families, 181 genera, and 189 species, with approximately 366 ASVs identified at the species level. Among these, all ASVs corresponding to potentially pathogenic organisms were filtered to create a secondary dataset, comprising 730,256 reads and 118 ASVs. This subset was distributed across five phyla, 10 classes, 14 families, 16 genera, and 22 species, from which 12 ASVs remained unclassified at the species level.

**Fig 2:**
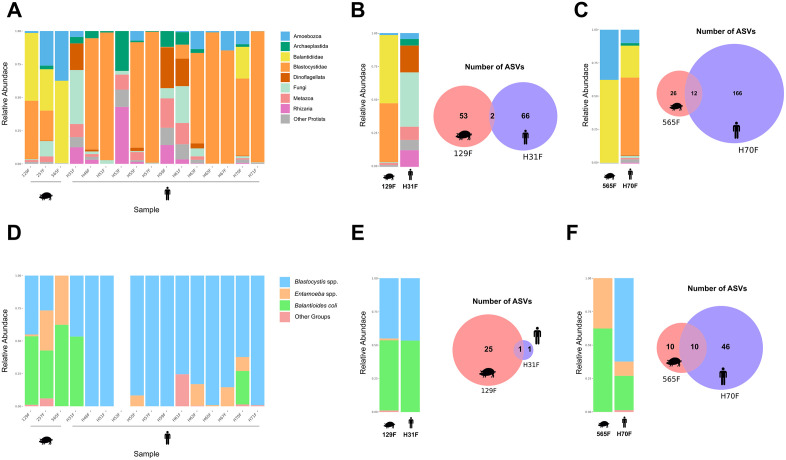
Comparison of eukaryotic and other taxonomic communities in humans and pigs via metabarcoding. (A) Relative abundance of all ASVs classified into major taxonomic groups. (B–C) Relative abundance of major groups and Venn diagrams showing shared and unique ASVs between pigs (129F in B; 565F in C) and humans (H31 in B; H70 in C), all positive for *B. coli* ASVs. (D) Relative abundance of ASVs assigned to potentially pathogenic organisms grouped into major categories. (E–F) Equivalent comparisons to B–C, restricted to potentially pathogenic groups, between pig–human pairs positive for *B. coli* (129F and H31 in E; 565F and H70 in F).

Analysis of the overall fecal microbiota community based on metabarcoding results revealed a wide diversity of taxa, with high abundances of eukaryotes from the family Blastocystidae, as well as members of the phylum Amoebozoa and the family Balantididae in both human and pig samples. Additionally, ASVs corresponding to other eukaryotic taxa and from other kingdoms were also detected (Fig 2A). Focusing specifically on ASVs from the family Balantididae, these were detected in the feces of two individuals, H31 and H70, both rural producers with direct contact with pigs—H31 from Silva Jardim and H70 from Nova Friburgo—as well as in the feces of pigs from their respective properties, pig 129F and pig 565F (Fig 2B and C).

A total of 68 ASVs were identified in H31 and 55 in pig 129F from the same property. Two ASVs were shared between these hosts, one belonging to the family Balantididae and the other to the kingdom Fungi. Only one ASV of the family Balantididae was detected in H31, which is not shown in the general community abundance plot due to its low frequency (eight reads). H31 exhibited lower eukaryotic taxon diversity compared to H70, with no ASVs from the family Blastocystidae detected (Fig 2B). In H70, 178 ASVs were observed, whereas 38 ASVs were detected in pig 565F. Of these, 12 ASVs were shared between both host species, belonging to the family Balantididae, the phylum Amoebozoa, and the kingdom Fungi. Additionally, shared ASVs from the family Blastocystidae, the phylum Dinoflagellata, and the kingdom Metazoa were detected in both hosts, although they were not highlighted in the relative abundance plot of pig 565F due to their low frequency (Fig 2C).

**Fig 3:**
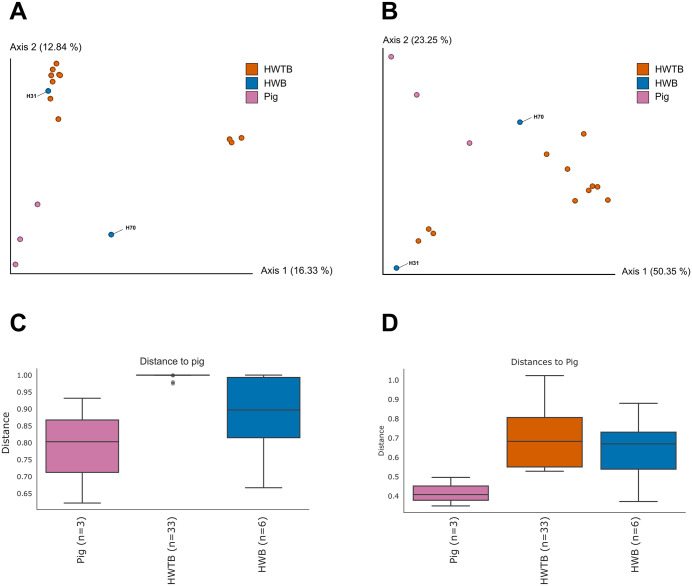
Fecal eukaryotic and other taxonomic communities in humans and pigs, stratified by *Balantioides coli.* (A–B) Principal Coordinates Analysis (PCoA) based on Bray–Curtis distances (A) and Weighted UniFrac distances (B), showing separation among pig samples, humans without *B. coli* ASVs (HWTB), and humans with *B. coli* ASVs (HWB, H31, and H70). (C–D) Boxplots of Bray–Curtis (C) and Weighted UniFrac (D) distances comparing human and pig samples.

The sampling depth used for analyses considering all identified ASVs was 43,773. Principal Coordinates Analysis (PCoA), based on Bray–Curtis (Fig 3A) and Weighted UniFrac (Fig 3B) metrics, revealed distinct patterns in the overall eukaryotic community structure among the evaluated groups: humans with *B. coli* ASVs (H70 and H31) (HWB), humans without ASVs of this parasite (HWTB), and pigs that also harbored *B. coli* ASVs. In both analyses, pig samples formed a cohesive cluster, whereas humans without *B. coli* ASVs remained separated from this cluster, showing greater dissimilarity relative to pigs. Conversely, human samples with *B. coli* ASVs displayed an intermediate distribution, with some points positioned closer to the pig group, suggesting higher similarity between these two groups.

The distance boxplots for pigs (Fig 3C and D) reinforced this observation. For the Bray–Curtis metric (Fig 3C), human samples without *B. coli* ASVs showed higher distances relative to pig samples, whereas the feces from humans H70 and H31 exhibited lower values, indicating greater compositional similarity. The same pattern was observed for the Weighted UniFrac metric (Fig 3D), which accounts for phylogenetic relationships, confirming the higher structural similarity between the feces of pigs and humans harboring confirmed *B. coli* ASVs.

Statistical analyses confirmed these patterns. In the overall analysis, significant differences were observed among the groups for both metrics (Bray-Curtis: Pseudo-F = 1.34, p = 0.021; Weighted UniFrac: Pseudo-F = 3.25, p = 0.007). Pairwise comparisons revealed that humans lacking *B. coli* ASVs differed significantly from pig fecal samples (Bray-Curtis: p = 0.004; Weighted UniFrac: p = 0.004), whereas feces from producers harboring *B. coli* ASVs did not differ significantly from pig samples (Bray-Curtis: p = 0.483; Weighted UniFrac: p = 0.289). Moreover, the Weighted UniFrac results indicated a trend toward separation between humans with and without the parasite ASVs (p = 0.088), suggesting that the presence of *B. coli* influences the phylogenetic structure of the human eukaryotic gut community.

Regarding potentially pathogenic eukaryotic parasites, high abundances of *Blastocystis* spp. ASVs were detected. These were present in human feces (12/13) and pig feces (2/3), in addition to *Entamoeba* spp. (5/13 and 3/3) and *B. coli* (2/13 and 3/3) in humans and pigs, respectively (Fig 2D).

A total of ten *B. coli* ASVs were recovered, six of which were identified in human feces. Among these, five ASVs were also detected in the feces of pigs from Nova Friburgo, Silva Jardim, and Saquarema, i.e., in the properties where the presence of the parasite’s DNA had been confirmed in human feces. Focusing on the eukaryotic microbiota composition of potentially pathogenic parasites in the pig producers H31 and H70, it was observed that both shared only *B. coli* ASVs with their respective pig, with this genetic variant being the sole ASV detected between producer H31 and pig 129F (Fig 2E). In contrast, in the fecal material of producer H70 and pig 565F, in addition to two shared *B. coli* ASVs, *Entamoeba* spp., *Blastocystis* spp., and other eukaryotes were also detected between these hosts (Fig 2F). Overall, 9,919 *B. coli* reads were identified in the fecal material of producer H70.

**Fig 4:**
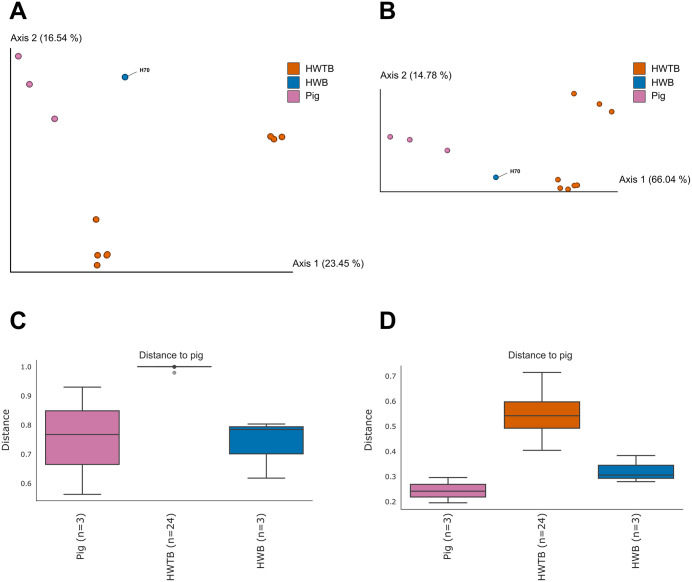
Potentially pathogenic eukaryotic parasites in feces of pigs and humans, stratified by *Balantioides coli* presence. (A–B) Principal Coordinates Analysis (PCoA) based on Bray–Curtis distances (A) and Weighted UniFrac distances (B), showing separation among pig samples, humans without *B. coli* ASVs (HWTB), and humans with *B. coli* ASVs (HWB, H31, and H70). (C–D) Boxplots of Bray–Curtis (C) and Weighted UniFrac (D) distances comparing human and pig samples.

Beta-diversity analysis was also conducted for the dataset consisting solely of potentially pathogenic parasite ASVs, using a sampling depth of 38,759. Due to this cutoff, samples H31, H53, H59, and H61 were excluded from diversity analyses because they had very low read counts. The Bray-Curtis metric (Fig 4A) revealed a clear separation between pig and human samples: humans without *B. coli* ASVs were more distant from the pig cluster, while the human sample with *B. coli* ASVs was positioned closer to the pig samples. This pattern was even more pronounced using the Weighted UniFrac metric (Fig 4B), where the human sample with the parasite ASVs grouped closely with the pig cluster.

Boxplots (Fig 4C and D) supported these observations: humans without *B. coli* ASVs showed greater distances relative to pigs, whereas the human sample with the ciliate ASVs displayed lower distances, indicating higher similarity with the pig samples. Statistical tests for Bray-Curtis were not significant globally (p = 0.055), but pairwise comparisons revealed a significant difference between human samples without *B. coli* ASVs and pig feces (p = 0.012). Weighted UniFrac analysis was highly significant (p = 0.005), confirming that humans lacking *B. coli* ASVs harbored a markedly distinct community of potentially pathogenic eukaryotes compared to pigs (p = 0.005) (Table 2).

**Table 2:** PERMANOVA results from dissimilarity analyses (Bray–Curtis and Weighted UniFrac) comparing eukaryotic community structure in fecal samples from pig producers and their household members, and pigs with *Balantioides coli.*

**Bray-Curtis Geral Comparation for All The ASVs**			**Weighted Unifrac Geral Comparation for All The ASVs**		
Pseudo-F	p-value		Pseudo-F	p-value	
1.342992	0.021*		3.247393	0.007*	
**Bray-Curtis Pair-Wise Comparation for All The ASVs**			**Weighted Unifrac Pair-Wise Comparation for All The ASVs**		
Pair	Pseudo-F	p-value	Pair	Pseudo-F	p-value
HWTB – HWB	0.876158	0.556	HWTB – HWB	1.5849	0.088
HWTB – Pig	1.913092	0.004*	HWTB – Pig	5.836805	0.004*
Pig – HWB	1.054665	0.483	Pig – HWB	1.467299	0.289
**Bray-Curtis Geral Comparation for Possible Parasites ASVs**			**Weighted Unifrac Geral Comparation for Possible Parasites ASVs**		
Pseudo-F	p-value		Pseudo-F	p-value	
1.376961	0.055		9.042682	0.005*	
**Bray-Curtis Pair-Wise Comparation for Possible Parasites ASVs**			**Weighted Unifrac Pair-Wise Comparation for Possible Parasites ASVs**		
Pair	Pseudo-F	p-value	Pair	Pseudo-F	p-value
HWTB – HWB	0.743546	0.658	HWTB – HWB	2.141571	0.241
HWTB – Pig	2.115773	0.012*	HWTB – Pig	17.223301	0.005*
Pig – HWB	0.892189	0.751	Pig – HWB	2.098291	0.232

ASVs: amplicon sequence variant; *p ≤ 0.05: significant; HWTB: human without *Balantioides coli* ASVs; HWB: human with *Balantioides coli* ASVs.

### Fecal samples from rural pig farmers and pigs positive for *B. coli*: Comparison of the associated unicellular prokaryotic microbiota

The same fecal samples analyzed for eukaryotes by metabarcoding were also assessed for their unicellular prokaryotic composition. Initial processing generated 1,278,967 reads. After primer removal and QIIME2 filtering, 1,273,379 reads remained and were clustered into 15,225 amplicon sequence variants (ASVs), classified into 57 phyla, 154 classes, 604 families, and more than 1,200 genera. ASVs corresponding to organisms associated with gastrointestinal eubiosis and potentially pathogenic taxa (grouped at the genus level) were filtered, resulting in a secondary dataset comprising 335,289 reads and 1,025 ASVs. This subset was distributed across four phyla, five classes, nine families, and approximately 19 genera.

**Fig 5:**
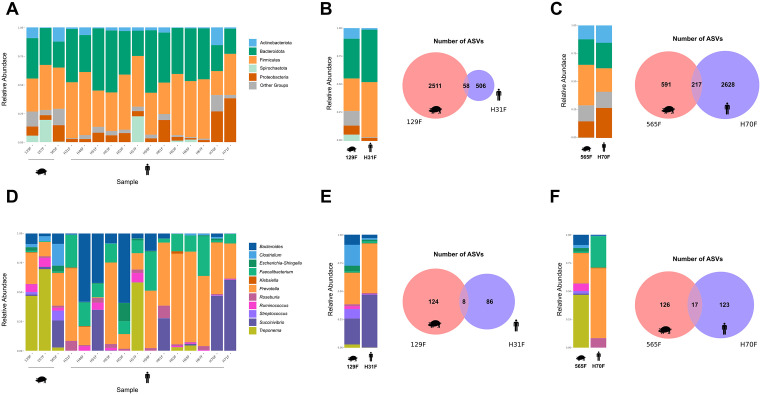
Comparison of prokaryotic fecal communities in humans and pigs recovered via metabarcoding analyses. (A) Relative abundance of all ASVs classified at the phylum level. (B–C) Relative abundance at the phylum level and Venn diagrams showing shared and unique ASVs between pigs (129F in B; 565F in C) and humans (H31 in B; H70 in C), all positive for *Balantioides coli*. (D) Relative abundance of ASVs belonging to two prokaryotic groups: taxa associated with gastrointestinal eubiosis and potentially pathogenic taxa, grouped at the genus level. (E–F) Equivalent to panels B–C, but restricted to these prokaryotic groups, comparing pig–human pairs positive for *B. coli* (129F and H31 in E; 565F and H70 in F).

Analysis of the relative abundance of the overall prokaryotic community revealed broad diversity. Firmicutes and Bacteroidota were the predominant phyla, while Proteobacteria showed notable abundance in some individuals (H71, H70, H61, H55, H51, 565F). Spirochaetota and Actinobacteriota appeared at lower frequencies and only in specific producers and pigs (Fig 5A).

A targeted analysis of the prokaryotic communities in the fecal samples of producers H31 and H70 and their pigs (129F and 565F), which also harbored Balantididae ASVs, identified 2,569 ASVs in pig 129F and 564 in its producer (H31). Of these, 58 ASVs were shared, mainly within Firmicutes, Actinobacteriota, Bacteroidota, and Proteobacteria. These phyla were the most abundant overall, while ASVs from other phyla were detected at lower proportions (Fig 5B). In pig 565F, 808 ASVs were detected compared with 2,845 in producer H70, of which 217 were unique to each host. The same dominant phyla shared by H31 and 129F were also present in H70 and 565F, although additional low-frequency taxa were observed among shared ASVs in the latter pair (Fig 5B and C). Analyses including all ASVs were conducted using a sampling depth of 43,904 sequences per sample to standardize effort across groups.

A focused analysis of prokaryotes associated with gastrointestinal eubiosis and potential pathogenicity showed that the most abundant ASVs in both producers and pigs belonged to the genera *Bacteroides* and *Prevotella*. Other genera, including *Escherichia–Shigella*, *Ruminococcus*, *Treponema*, *Roseburia*, and *Klebsiella*, were detected at lower proportions in both hosts. *Clostridium* and *Streptococcus* were more abundant in pigs, whereas *Succinivibrio* and *Faecalibacterium* were more prevalent in humans (Fig 5D).

Among producers positive for *B. coli* ASVs, H31 and H70 harbored 94 and 140 prokaryotic ASVs, respectively. Their pigs (129F and 565F) harbored 132 and 143 ASVs, respectively. The overlap between hosts included eight shared ASVs in the H31–129F pair and 17 in the H70–565F pair. Shared genera included *Prevotella*, *Faecalibacterium*, *Clostridium*, *Escherichia–Shigella*, *Klebsiella*, *Roseburia*, and *Bacteroides*. In H70 and 565F, *Streptococcus* was also detected (Fig 5E and F). For analyses restricted to eubiosis-associated and potentially pathogenic taxa, a sampling depth of 11,965 sequences per sample was applied. As a result of this cutoff, samples 565F and 129F were excluded because of low counts of ASVs of interest.

**Fig 6:**
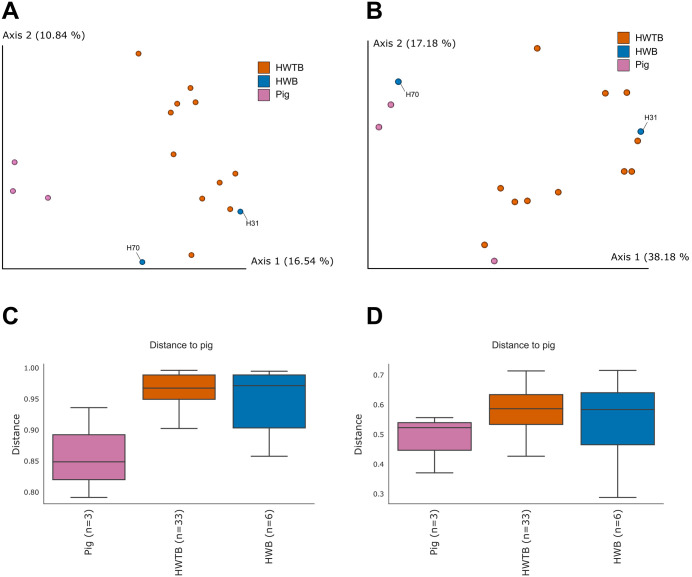
Prokaryotic fecal communities in pig farmers, household members, and pigs, according to *Balantioides coli* presence. (A–B) Principal coordinates analysis (PCoA) based on Bray–Curtis (A) and Weighted UniFrac (B) distances, showing the separation among pig samples, humans without *B. coli* ASVs (HWTB), and humans with *B. coli* ASVs (HWB, H31, and H70). (C–D) Boxplots of Bray–Curtis (C) and Weighted UniFrac (D) distances comparing human and pig samples.

Principal coordinates analysis (PCoA) based on Bray–Curtis dissimilarities (Fig 6A) showed that humans without *B. coli* ASVs formed a compact cluster clearly separated from pigs harboring Ciliophora ASVs. In contrast, humans with *B. coli* ASVs were more dispersed, with H70 positioned closer to the pig microbiota. Average distance analysis for pigs (Fig 6C) confirmed this pattern: Bray–Curtis values indicated shorter distances between pigs and humans with *B. coli* ASVs compared with humans lacking these ASVs, who consistently showed higher dissimilarity.

Weighted UniFrac ordination (Fig 6B) further supported these results. Pigs (129F and 565F) grouped closer to producer H70, while humans without *B. coli* ASVs formed a cohesive cluster with pig 257F. Boxplots (Fig 6D) also reflected this trend: humans without *B. coli* ASVs exhibited greater distances from pigs, whereas humans with the parasite clustered closer to them.

PERMANOVA post hoc tests corroborated these observations. Significant differences were found among groups for both Bray–Curtis (Pseudo-F = 1.71, p = 0.001) and Weighted UniFrac (Pseudo-F = 2.06, p = 0.019). Pairwise comparisons showed significant differences between producers without *B. coli* ASVs and pigs (Bray–Curtis: p = 0.002; UniFrac: p = 0.004), but no significant differences between producers harboring *B. coli* ASVs and pigs (Bray–Curtis: p = 0.097; UniFrac: p = 0.613) (Table 3).

**Table 3:** Results of PERMANOVA tests applied to dissimilarity analyses (Bray–Curtis and Weighted UniFrac) comparing the prokaryotic community structure in fecal samples from pig farmers and their household members, and pigs with *Balantioides coli.*

**Bray-Curtis Geral Comparation for All The ASVs**			**Weighted Unifrac Geral Comparation for All The ASVs**		
Pseudo-F	p-value		Pseudo-F	p-value	
1.709965	0.001*		2.062300	0.019*	
**Bray-Curtis Pair-Wise Comparation for All The ASVs**			**Weighted Unifrac Pair-Wise Comparation for All The ASVs**		
Pair	Pseudo-F	p-value	Pair	Pseudo-F	p-value
HWTB – HWB	0.967195	0.533	HWTB – HWB	0.747392	0.634
HWTB – Pig	2.424788	0.002*	HWTB – Pig	3.775612	0.004*
Pig – HWB	1.609802	0.097	Pig – HWB	1.077045	0.613
**Bray-Curtis Geral Comparation for Possible Parasites ASVs**			**Weighted Unifrac Geral Comparation for Possible Parasites ASVs**		
Pseudo-F	p-value		Pseudo-F	p-value	
1.326.666	0.09		3.437541	0.086	
**Bray-Curtis Pair-Wise Comparation for Possible Parasites ASVs**			**Weighted Unifrac Pair-Wise Comparation for Possible Parasites ASVs**		
Pair	Pseudo-F	p-value	Pair	Pseudo-F	p-value
HWTB – HWB	0.834181	0.739	HWTB – HWB	0.373912	0.873
HWTB – Pig	1.737044	0.08	HWTB – Pig	6.005879	0.081
Pig – HWB	2.148201	0.324	Pig – HWB	635.399	0.344

ASVs: amplicon sequence variant; *p ≤ 0.05: significant; HWTB: human without *Balantioides coli* ASVs; HWB: human with *Balantioides coli* ASVs.

**Fig 7:**
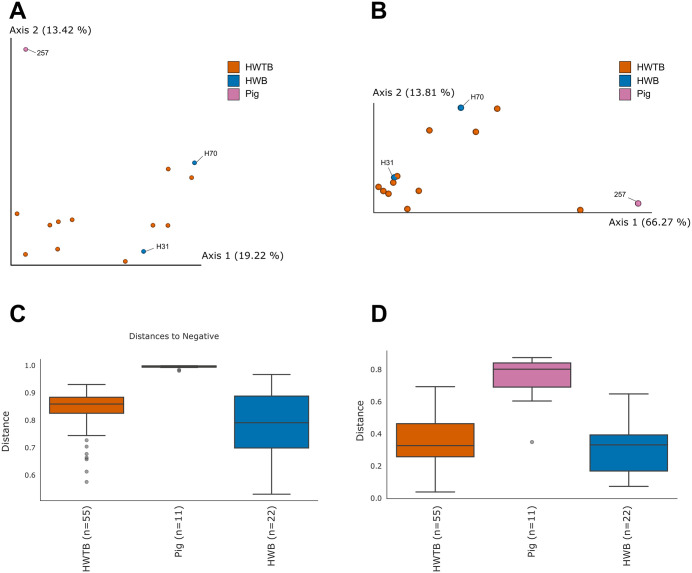
Prokaryotic fecal communities, including potentially pathogenic taxa, in humans and pigs stratified by *Balantioides coli.* (A–B) Principal coordinates analysis (PCoA) based on Bray–Curtis (A) and Weighted UniFrac (B) distances, showing the separation among the positive pig sample, humans without *B. coli* ASVs (HWTB), and humans with *B. coli* ASVs (HWB, H31, and H70). (C–D) Boxplots of Bray–Curtis (C) and Weighted UniFrac (D) distances comparing human samples with the positive pig sample.

Scatterplots assessing eubiosis-associated and potentially pathogenic prokaryotes (Fig 7A and B) showed no clearly defined clusters among humans with *B. coli* ASVs, humans without ASVs, and pigs. Nonetheless, a trend toward separation between pig and human microbiota was observed, particularly relative to humans lacking *B. coli*. Distance boxplots (Fig 7C and D) confirmed this trend: individuals without *B. coli* ASVs exhibited higher median distances from pigs compared with producers harboring *B. coli* ASVs. Post hoc tests revealed no statistically significant global differences. However, some p-values exhibited a marginal trend toward significance, particularly when comparing pigs with humans lacking *B. coli* ASVs (Table 3).

## Discussion

In this study, the occurrence of *Balantioides coli* in fecal samples from rural pig farmers was investigated using a combination of microscopic and molecular techniques, including polymerase chain reaction (PCR) followed by Sanger sequencing. A positivity rate of 14.9% was observed among rural farmers, with a higher prevalence in the state of Rio de Janeiro. Higher frequencies have been documented in Brazilian schoolchildren, including children and adults with intellectual and/or multiple disabilities—6/28 (21.4%) in Espírito Santo, detected by spontaneous sedimentation, and 10/42 (23.8%) in children from a rural area in Paraná, identified by flotation and sedimentation microscopy [40,41]. Even greater prevalence has been reported in children with intestinal disorders in Iraq, reaching 36.3% (8/100) [42].

By contrast, lower frequencies of *B. coli* have been reported in Brazil, with prevalence ranging from 0.74% to 2.9% in adolescents and adults attending a public hospital in Rio de Janeiro, children and adolescents in Pará, Bahia, and Santa Catarina, indigenous populations in Mato Grosso, and patients with HIV in Pernambuco [43–48]. Similarly low frequencies (0.5%–1.8%) have been described in Bolivia, Argentina, and Panama [49–52], in India and Bangladesh (0.2%–6.7%) [53–55], and in African countries including Angola, Burkina Faso, Ethiopia, Nigeria, and Ghana (0.3%–10.4%) [56–63].

The wide range of reported frequencies may reflect differences in sample size, geography, host populations, and especially the diagnostic techniques employed. Importantly, most previous studies relied exclusively on microscopy. In the present study, no forms compatible with *B. coli* were detected by microscopy in farmers’ feces, consistent with prior studies from Brazil and Venezuela [14–16,64]. In general, trophozoites are the diagnostic stage is more than 80% of human balantidiasis cases, and their detection depends on fresh stool examination [4,65–67]. Consequently, although direct fecal examination was performed, the samples were no longer fresh when analyzed in the laboratory. This limitation, combined with the intermittent shedding of parasitic forms, may have hindered the microscopic diagnosis of the parasite in human samples. Therefore, the detection of *B. coli* in the feces of pig farmers was only possible through molecular techniques, including polymerase chain reaction (PCR) using primers targeting the ITS1–5.8S–ITS2 region specific to the Ciliophora group, followed by Sanger sequencing. These molecular approaches are well established in the literature for the specific diagnosis of this parasite [26,27,68–72].

Although Brazil has historically reported numerous cases of human balantidiasis, all previous studies relied exclusively on microscopy [4,12]. Therefore, this is the first study to identify *B. coli* in human samples using molecular tools within Brazilian territory. For the Ciliophora group, molecular diagnosis is essential for reliable taxonomic identification, given that this group comprises a large number of morphologically similar species [73]. Unfortunately, microscopists in epidemiological parasitology studies and clinical case reports often immediately classify trophozoites and cysts of the Ciliophora group as *B. coli*, a premature diagnostic assumption [4]. Moreover, molecular techniques enhance diagnostic sensitivity, as the presence of intact parasitic forms in biological material is not required.

This study included all pig farmers and household members present during farm visits (47 individuals). For pigs, a single fecal sample per farm was collected to enable direct comparisons with farmers, but financial constraints limited the number of animal samples. The molecular diagnosis of *B. coli* was primarily observed in fecal samples from participants who directly handled pigs on both family-based and industrial farms located in the state of Rio de Janeiro. This parasite was also identified through microscopic and molecular techniques in pig fecal samples from all participating farms. These findings underscore the importance of pigs as a source of *B. coli* infection and highlight direct animal handling as a risk factor for human balantidiasis. The role of pigs as a reservoir for *B. coli* infection in humans is consistent with previous findings from our research group, as well as with reports from other authors [4,12,74].

Transmission of this parasite may have occurred due to the incomplete use of personal protective equipment (PPE). During questionnaire administration, most farmers considered PPE to consist only of closed shoes and/or boots and long pants. The use of masks, gloves, or protective eyewear was neither reported nor observed in any property. Such practices may have facilitated passive oral transmission of the parasite through ingestion of aerosols generated from pig organic matter or via hand-to-mouth contact after handling the animals and/or their excreta. Furthermore, the results of the present study demonstrate the occurrence of zoonotic transmission of *B. coli* in Brazil. Therefore, diagnostic tools with high specificity and sensitivity for this parasite, including molecular approaches, should always be employed when analyzing samples from pig farmers, as they are continuously exposed to this parasite.

Although the sample size of participants in this study was relatively small, it is important to note that the participation rate was high, reaching 92% (47/51). Most participants, including those with fecal samples positive for *B. coli*, were adult males. Similar findings have been reported among pig farmers in Ghana, as well as in both urban and rural areas of Ethiopia [61–63]. In general, males constitute the primary workforce in pig farming in Brazil, directly handling the animals and therefore being at greater risk of exposure [16]. Nevertheless, molecular evidence of *B. coli* was also identified in the fecal material of a female participant from a property in Saquarema, Rio de Janeiro (H46), who was a family member of the farmer and had no direct contact with the animals. This observation further highlights the potential for indirect transmission of this parasitic agent.

It is well established that the clothing worn by pig farmers who directly handle the animals can play an important role in the indirect transmission of the parasite to other household members, including women and children, who do not work with pigs. This issue regarding the use, changing and laundering of work clothing by rural pig farmers has also been identified as a key factor in the transmission of *B. coli* to other family members on farms in Ghana [63]. Work attire can contaminate the household environment and the clothing of other individuals when washed together. Unfortunately, this issue was also observed during fieldwork on pig farms in Rio de Janeiro and Minas Gerais. However, the majority of participants reported practices that differed from those actually observed by the field team during questionnaire administration.

Most pig farms included in this study were located in the peridomestic area of the rural producers’ residences. This setting encompassed individuals who tested positive for *B. coli* in their fecal samples, such as the family farm in Saquarema, as well as the industrial-type farm in Nova Friburgo. Among the industrial farms included in this study, the Nova Friburgo farm was the only one with employee housing situated around the pig facilities. The consumption of water from springs and wells, as well as vegetables grown in peridomestic gardens, should also be highlighted as potential indirect sources of parasite infection. In general, untreated water has been consistently identified in the literature as one of the main sources of intestinal parasitic infections, including *B. coli*, along with the ingestion of contaminated vegetables [41,48,50,58,61,75–78]. Thus, the close physical proximity of pig facilities to untreated water sources and vegetable crops, as observed on the farms in this study, may have favored environmental contamination with parasite cysts, thereby facilitating transmission to humans.

Among the individuals diagnosed with *B. coli*, only two reported diarrhea, and one of these also reported blood in the stool—clinical manifestations consistent with human balantidiasis. Nevertheless, most individuals reported no gastrointestinal disturbances. These findings highlight the possibility of asymptomatic cases, chronic infections with mild and sporadic clinical manifestations that go unnoticed by infected individuals, or even reporting biases due to embarrassment. In addition, it cannot be excluded that molecular diagnosis may detect convalescent cases — i.e., infections that are no longer active and in which the parasite is no longer replicating — or accidental ingestion of parasite-derived DNA fragments.

Overall, *B. coli* nucleotide sequences compatible with types B0, A0, and atypical sequences not conforming to the classical genetic variant classification proposed by Ponce-Gordo et al. [68] were generated from the fecal material of family farmers. In pig fecal samples, a predominance of B0 type variants was observed. At the Nova Friburgo property, human participants with molecular evidence of the parasite (H53, H63, and H70) harbored the same *B. coli* variant type identified in the pig fecal sample from that farm, namely B0 (565F). Among the nucleotide sequences consulted and used as references for B0 and A0 types, the variants have previously been reported infecting other host species, particularly domestic and feral pigs in Brazil, as well as domestic pigs in Italy, Spain, China, and South Korea [26,71,79–82].

*Balantioides coli* sequences of types A0 and B0 have also been reported in fecal material from neotropical and Old World non-human primates in Brazil, respectively [72,83]. Additionally, type A0 nucleotide sequences have been documented in guinea pigs in China [84]. Furthermore, two samples contained *B. coli* fragments that could not be classified and were therefore designated as atypical. A similar classification—that is, sequences not conforming to previously established patterns—was recently reported in fecal samples from the largest neotropical non-human primate in a Brazilian biome [83]. The presence of additional variants at lower frequency suggests the introduction or persistence of less common lineages, possibly associated with conjugation events, gene flow, or circulation in under-studied host species.

The absence of exclusive phylogenetic clustering for sequences derived from humans and pigs indicates that these hosts share similar variants, thereby reinforcing the zoonotic potential of the parasite. This genetic proximity with isolates described in non-human primates, rodents, and other animals supports the hypothesis of interspecies transmission, underscoring the importance of molecular surveillance across diverse animal populations. The results demonstrate that both family-based and industrial pig production expose humans to *B. coli* parasitism and allow the circulation of variants with distinct genetic profiles, reflecting the high adaptive plasticity of *B. coli*. This scenario is highly relevant to public health, as genetically divergent variants may differ in pathogenicity, transmission potential, and response to control measures, highlighting the urgent need for further investigation.

A search of public molecular databases revealed only two *B. coli* nucleotide sequences from human samples in Bolivia, both belonging to type A (one A0 and one A2) [68]. Thus, the present study represents only the second molecular epidemiology-focused investigation to provide data from potential human infections with this parasite. Importantly, this study expanded current knowledge of the genetic variants present, as both type B0 and atypical variants not previously reported in human fecal material were identified.

Metabarcoding analysis revealed the presence of eukaryotic taxa in human fecal samples that were not detected using conventional microscopy-based parasitological techniques. This outcome reflects the broad coverage of the molecular marker employed—the V4 fragment of the 18S rRNA gene—which is highly conserved and therefore capable of amplifying DNA from a wide diversity of eukaryotes within a single sample. This methodological strategy not only enhanced the detection of potentially pathogenic organisms but also enabled the identification of potential mixed infections involving *B. coli* and other eukaryotes. Notably, this is the first study to explore, from a molecular epidemiology perspective, the coexistence of *B. coli* with other eukaryotes using metabarcoding approaches.

However, for the diagnosis of *B. coli*, conventional PCR followed by Sanger sequencing targeting the ITS1–5.8S–ITS2 region revealed a higher frequency of positive human samples compared with metabarcoding of the V4 region of the 18S rRNA gene. A similar finding was reported in an investigation of *Giardia duodenalis* in patients from a hospital in Algeria, where real-time PCR targeting the 18S rRNA detected a significantly higher positivity rate (60 cases; 66%) compared with metabarcoding of the V3–V4 regions (six cases; 6.6%) [85]. The greater efficiency observed with Sanger sequencing may be linked to the specificity of the ITS target, which is directed at the phylum Ciliophora, to which *B. coli* belongs, thereby enhancing its detection. In contrast, metabarcoding using the V4 region, being a highly conserved eukaryotic marker, promotes competitive amplification of DNA from other organisms, often more abundant in the samples and constituting the host intestinal microbiota, as has been reported for *Blastocystis* colonization [18,85,86].

It should also be noted that while universal primers provide broad taxonomic coverage, they can simultaneously amplify host DNA and dietary DNA present in fecal samples. This can result in a masking effect in which the target parasite DNA is obscured by more abundant host or non-target DNA, potentially leading to underestimation of parasite burden or the loss of low-abundance parasites [87]. Such a situation may have occurred in the present study, since humans are not the primary hosts of *B. coli*, making its detection particularly challenging.

In the fecal samples of the two rural producers (H31 and H70) and their respective pigs (129F and 565F), shared ASVs of *B. coli* were identified, along with ASVs of other eukaryotic taxa, most notably *Blastocystis* spp. and *Entamoeba* spp., in the feces of human H70 and pig 565F. This sharing of *B. coli* ASVs as well as *Blastocystis* spp. and *Entamoeba* spp. ASVs between human and pig samples, was corroborated by statistical analyses demonstrating consistent similarity in the eukaryotic microbiota of human and animal feces. These findings suggest that *B. coli* should not be considered solely as an isolated zoonotic infection but rather as part of a broader context of zoonotic coinfections. This hypothesis is further supported by the fact that *Blastocystis* spp. and certain *Entamoeba* species that infect pigs also exhibit zoonotic potential and can be transmitted to humans. Thus, the sharing of ASVs indicates the possibility of coexistence or competition for ecological niches between *B. coli* and other intestinal eukaryotes, which may explain the greater similarity observed between parasitic communities in exposed humans and pigs, the latter recognized as the natural hosts of *B. coli*.

It is noteworthy that *Blastocystis* spp. was the most abundant eukaryote detected in the fecal samples of rural producers. Overall, the literature remains inconclusive regarding the biological role of this organism, with debate as to whether it should be considered pathogenic, commensal, or opportunistic—sometimes implicating it and at other times exonerating it as a causative agent of gastrointestinal disorders [88,89]. However, more recent studies have associated the presence of *Blastocystis* spp. with eubiosis, suggesting that this organism may promote increased richness of beneficial bacterial taxa, thereby contributing to intestinal microbiota balance and mucosal homeostasis [85,86,90,91].

In addition, xenic culture studies have reported that the isolation and *in vitro* maintenance of *B. coli* may be hindered by the concomitant growth of *Blastocystis* spp. [71]. Although the interactions between these two eukaryotes remain poorly understood, the high abundance of *Blastocystis* spp. in the fecal samples of the rural producers investigated here may have limited the colonization of *B. coli* in the large intestine, thereby favoring asymptomatic infections, as observed in producers H31 and H70.

Analysis of the intestinal microbiota of rural producers with and without *B. coli* ASVs, as well as pigs, revealed a highly diverse bacterial community dominated by the phyla Firmicutes and Bacteroidota, typical of mammalian gut microbiota. These results are consistent with previous reports in nursery-phase pigs in intensive production systems in China, both infected and uninfected with *B. coli* [19,20]. Members of the phylum Firmicutes, such as *Roseburia, Clostridium, Faecalibacterium,* and *Ruminococcus,* are known to produce short-chain fatty acids, which regulate intestinal immune responses and inhibit the activity of several opportunistic pathogens [86].

Moreover, gastrointestinal species of Bacteroidota also produce short-chain fatty acids, such as succinate, propionate, acetate, and butyrate, which act as key metabolic end-products [92]. Among these, butyrate plays a central role, serving as the primary energy source for colonic epithelial cells. This fatty acid is metabolized through the β-oxidation pathway, resulting in ATP generation. This process consumes molecular oxygen and consequently reduces luminal oxygen concentration, thereby favoring eubiosis [86]. Thus, the abundance of Firmicutes and Bacteroidota observed in the fecal samples of rural producers and pigs in the present study contributes to the maintenance of intestinal microbial homeostasis; however, this same stable, low-oxygen microenvironment may also have favored the colonization of *B. coli*, a parasite considered a facultative anaerobe.

Analysis of the prokaryotic population revealed several shared bacterial ASVs between the fecal samples of *B. coli*-positive rural producers (H31 and H70) and their pigs (129F and 565F). In the human–pig pairs where *B. coli* ASVs were detected, shared bacterial genera included *Prevotella, Faecalibacterium, Clostridium, Escherichia-Shigella, Klebsiella, Roseburia,* and *Bacteroides.* This core bacterial community may play a central role in modulating intestinal immune responses and maintaining conditions favorable to protozoan persistence. Previous studies have shown that high abundance of *Prevotella*, particularly *P. copri*, a pro-inflammatory bacterium, is associated with increased susceptibility to intestinal colonization by *Entamoeba histolytica* in children in Bangladesh [91,93,94]. Considering that *E. histolytica* and *B. coli* share similar ecological niches—both colonizing the large intestine and capable of invading the intestinal mucosa—it is plausible to suggest that the presence of *P. copri* may also have facilitated the establishment or persistence of the ciliate.

In addition to *Prevotella*., ASVs of potentially pathogenic bacterial taxa were also concomitantly identified in the fecal samples of pigs and their caretakers harboring *B. coli* ASVs, notably the genera *Escherichia-Shigella* and *Klebsiella* from the family Enterobacteriaceae. Several *in vitro* studies have demonstrated that the presence of enteropathogenic bacteria can potentiate the pathogenic behavior of *E. histolytica*. For example, interactions with virulent bacteria such as enteropathogenic *Escherichia coli*, *Shigella dysenteriae*, or *Clostridium symbiosum* can enhance the activity of the parasite’s cysteine proteases, its cytopathic effect, erythrophagocytosis, hemolytic activity, and the host’s proinflammatory cytokine secretion [95]. For *B. coli*, however, the true relevance of this bacterial group in colonization success remains unclear. Nonetheless, a similar pattern of bacterial colonization, as reported in the present study, has been described in young diarrheic pigs infected with *B. coli* in China [19,20]. The concurrent detection of *Escherichia-Shigella* and *Klebsiella* in both infected pig fecal samples and in producers positive for *B. coli* ASVs reinforces the hypothesis of a zoonotic risk interface, particularly in settings involving direct contact and inadequate sanitary management. It is also noteworthy that the transmission route for these enterobacteria and the ciliate is shared, occurring via the fecal–oral pathway, which facilitates bacteria–eukaryote coinfections. Furthermore, the possibility that these prokaryotes may be carried as intracellular symbionts, residing within vacuoles in the cytoplasm of the parasite [96], cannot be excluded, which could contribute to the joint maintenance and dissemination of these agents within the host intestine.

## Conclusion

By integrating multiple laboratory tools, this study advances the understanding of the epidemiology of *B. coli* by providing the first molecular evidence of its occurrence in humans in Brazil, revealing shared variants between rural pig producers and pigs, and confirming the role of pigs as an important reservoir. The integration of molecular analyses with microbiota profiling demonstrated that the prokaryotic and eukaryotic communities present in the feces of *B. coli*-positive rural producers and their pigs exhibited remarkable similarities, which may be relevant for the colonization of this parasite, further supporting the zoonotic transmission of this ciliate and other infectious-parasitic agents. From a broader perspective, these findings underscore the need for strategies aligned with the One Health concept, integrating molecular surveillance, good management practices, improvements in sanitation, and monitoring of the intestinal microbiota as fundamental measures to reduce zoonotic risk and to better understand the true impact of *B. coli* at the human–animal–environment interface.

## Supporting information

S1 Table Information on the samples included in the study, including sample identification, country of origin, host species (human or pig), farm (family or industrial), and GenBank accession number. (DOCX)
