# Supplementary material for: One health approach to Balantioides coli: Molecular and metabarcoding evidence of zoonotic transmission
Source: PLoS One. 2026 Jan 5;21(1):e0338487. doi: 10.1371/journal.pone.0338487 (PMC12768370; doi:10.1371/journal.pone.0338487)
Supplement: S1 Table — (DOCX) [file pone.0338487.s001.docx]

| **Sample** | **Country** | **Host** | **Farm type** | **GenBank accession** |
| --- | --- | --- | --- | --- |
| H31 | Brazil | *Homo sapiens* | Family farm | PV706828 |
| H46 | Brazil | *Homo sapiens* | Family farm | PV706829 |
| H51 | Brazil | *Homo sapiens* | Family farm | PV706830 |
| H53 | Brazil | *Homo sapiens* | Industrial farm | PV706831 |
| H61 | Brazil | *Homo sapiens* | Industrial farm | PV706832 |
| H63 | Brazil | *Homo sapiens* | Industrial farm | PV706833 |
| H70 | Brazil | *Homo sapiens* | Industrial farm | PV706834 |
| P18 | Brazil | *Sus scrofa* | Industrial farm | PV706836 |
| P71 | Brazil | *Sus scrofa* | Family farm | PV706840 |
| P77 | Brazil | *Sus scrofa* | Family farm | PV706842 |
| P82 | Brazil | *Sus scrofa* | Family farm | PV706844 |
| P119 | Brazil | *Sus scrofa* | Family farm | PV706846 |
| P129 | Brazil | *Sus scrofa* | Family farm | PV706848 |
| P157 | Brazil | *Sus scrofa* | Family farm | PV706852 |
| P158 | Brazil | *Sus scrofa* | Family farm | PV706854 |
| P181 | Brazil | *Sus scrofa* | Family farm | PV706858 |
| P257 | Brazil | *Sus scrofa* | Family farm | PV706862 |
| P565 | Brazil | *Sus scrofa* | Industrial farm | PV706879 |
| P717 | Brazil | *Sus scrofa* | Family farm | PV706883 |
| P745 | Brazil | *Sus scrofa* | Family farm | PV706887 |
| P852 | Brazil | *Sus scrofa* | Industrial farm | PV706900 |
| P927 | Brazil | *Sus scrofa* | Industrial farm | PV706906 |
